# Time-of-Flight Imaging in Fog Using Polarization Phasor Imaging

**DOI:** 10.3390/s22093159

**Published:** 2022-04-20

**Authors:** Yixin Zhang, Xia Wang, Yuwei Zhao, Yujie Fang

**Affiliations:** 1 Key Laboratory of Photoelectronic Imaging Technology and System, Ministry of Education, School of Optics and Photonics, Beijing Institute of Technology, Beijing 100081, China; 3120190601@bit.edu.cn (Y.Z.); 3120200608@bit.edu.cn (Y.Z.); 12242@bitzh.edu.cn (Y.F.); 2 Beijing Institute of Technology, Zhuhai 519000, China

**Keywords:** time-of-flight camera, polarization defogging, phasor imaging, multipath interference, image recovery

## Abstract

Due to the light scattered by atmospheric aerosols, the amplitude image contrast is degraded and the depth measurement is greatly distorted for time-of-flight (ToF) imaging in fog. The problem limits ToF imaging to be applied in outdoor settings, such as autonomous driving. To improve the quality of the images captured by ToF cameras, we propose a polarization phasor imaging method for image recovery in foggy scenes. In this paper, optical polarimetric defogging is introduced into ToF phasor imaging, and the degree of polarization phasor is proposed to estimate the scattering component. A polarization phasor imaging model is established, aiming at separating the target component from the signal received by ToF cameras to recover the amplitude and depth information. The effectiveness of this method is confirmed by several experiments with artificial fog, and the experimental results demonstrate that the proposed method significantly improves the image quality, with robustness in different thicknesses of fog.

## 1. Introduction

**Figure 1:**
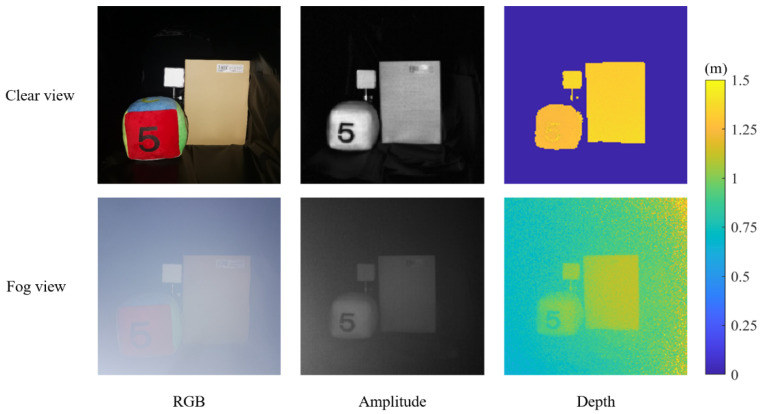
Amplitude and depth measurements in clear (upper) and foggy scenes (lower) using CW-ToF cameras.

Time-of-flight (ToF) imaging is an active depth-sensing technology with the advantages of a compact structure, low cost, and real-time image capture [1]. It is widely used in many fields, such as autonomous driving [2], machine vision [3], and human–computer interaction [4]. The continuous-wave ToF (CW-ToF) camera is a common depth-sensing camera, which indirectly acquires depth images by calculating the phase difference between sent and received signals [5], simultaneously capturing amplitude images of a scene. When a CW-ToF camera is used in foggy scenes, the contrast of the amplitude image is degraded and the depth image is greatly distorted, which limits the ToF camera to be applied in outdoor settings, as shown in Figure 1. Therefore, it is necessary to improve the quality of the images captured by a ToF camera in fog.

In a foggy scene, a CW-ToF camera receives the summation of reflected light from the target and scattering light caused by atmospheric aerosols. The light scattered in fog causes the multipath interference (MPI), which refers to the fact that a single pixel of the ToF camera receives multipath lights from the scene [6], leading to the significant error of depth measurements. Various studies have been implemented for MPI correction, such as mixed pixel restoration [7,8], multiple frequency measurements [9,10,11,12], compressed sensing [13,14], and deep learning [15,16,17]. In this work, we only consider the MPI caused by scattering media. To correct MPI generated by scattering media, many methods have been studied and developed, including convolutional sparse coding [18], data-driven prior [19], temporal properties [20], iterative optimization algorithms [21,22], image filtering [23], polarization properties of light [24,25], and phasor imaging [26,27]. Kijima D. et al. used multiple time-gating to estimate the scattering property of fog with a short-pulse ToF camera, for reconstructing the depth and intensity of a scene [20], which is not applicable to CW-ToF cameras. Wu R. et al. proposed the transient degree of polarization [24] and used the polarization properties of light to recover transient images in scattering scenes [25]. Transient images record the propagation of light through a scene with rich information [28], but they require hardware modifications or complex computation processes to accurately recover the time domain response [28,29,30,31]. Phasor imaging only recovers the depth information in the scattering scene by high-frequency [26] or multiple frequency measurements [27]. Our goal is to simultaneously recover the depth and amplitude information from a CW-ToF camera in the scattering scene, without restoring the transient images.

Optical polarimetric defogging is a powerful method to enhance visibility in fog, which uses the difference of polarization properties between the reflected and scattered light [32]. It can be divided into passive polarization defogging [33,34,35,36] and active polarization defogging [37,38,39,40,41], with good performance in visibility enhancement for RGB images. The effect of fog on amplitude images captured by a CW-ToF camera is different from that on RGB images, which follows the regularity of MPI. Therefore, optical polarimetric defogging cannot directly be used for restoring the amplitude image. Considering the principle of ToF imaging, we introduce polarization-based defogging to phasor imaging for improving the quality of images from a CW-ToF camera.

In this paper, we propose a polarization phasor imaging method for simultaneously recovering the depth and amplitude information of ToF imaging in fog, and establish the corresponding imaging model based on the polarization properties of light. The model estimates the scattering component using the degree of polarization phasor, and separates the target component to recover the depth and amplitude images. The main contributions of this work are:

- Firstly, we introduce optical polarimetric defogging to ToF phasor imaging, expanding the application of polarization defogging.
- Secondly, we define the degree of polarization phasor for describing the scattering effect for ToF imaging.
- Finally, we establish a polarization phasor imaging model for recovering amplitude and depth images in the foggy scenes by estimating the scattering component.

The remainder of this paper is organized as follows: Section 2 describes the phasor representation and models polarization phasor imaging; Section 3 verifies the effectiveness of the method through experiments; Section 4 discusses the limitations and potential future works; and Section 5 presents the conclusions.

## 2. The Polarization Phasor Imaging Method

**Figure 2:**
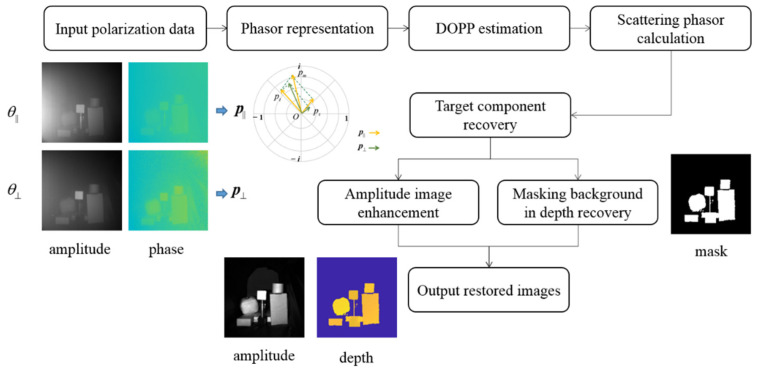
Pipeline for the polarization phasor imaging method. DOPP is an abbreviation for degree of polarization phasor.

The polarization phasor imaging method processes and analyzes polarization data in polar coordinates. First, a group of polarization images is captured at two orthogonal orientations of the linear polarization analyzer. The amplitude and depth captured at the same orientation are combined to form a phasor. Afterwards, the background is selected to estimate the degree of polarization phasor of the scattering component. After obtaining the scattering component, the amplitude and depth of the target component can be recovered. For a better performance, the image enhancement is applied in the amplitude, and masking background is used in the depth image. The pipeline of the proposed method is shown in Figure 2, and the details are discussed below.

### 2.1. Polarization Phasor Representation

**Figure 3:**
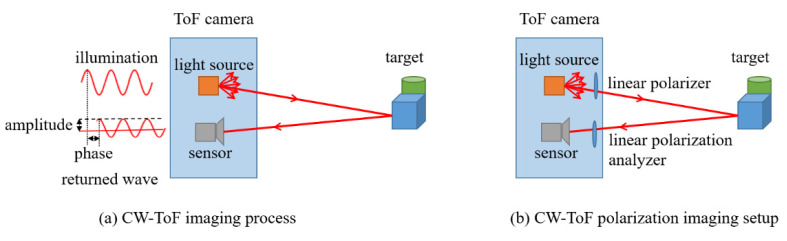
The CW-ToF imaging process and polarization imaging setup. (**a**) The light of a CW-ToF camera is modulated with continuous wave, and a CW-ToF camera simultaneously acquires the amplitude and depth of a scene. (**b**) The linear polarizers are added in CW-ToF imaging system, which constitutes the polarization imaging system.

A CW-ToF camera illuminates the scene with amplitude modulated light, and senses the depth information by calculating the phase shift between the sent and received signals. The amplitude image captured by a CW-ToF camera records the intensity information returned from the scene within a certain integration time, as shown in Figure 3a. In this work, the illumination beam of the ToF camera is polarized by a linear polarizer in front of the light source, and the images are captured from the sensor through a linear polarization analyzer, in Figure 3b.

**Figure 4:**
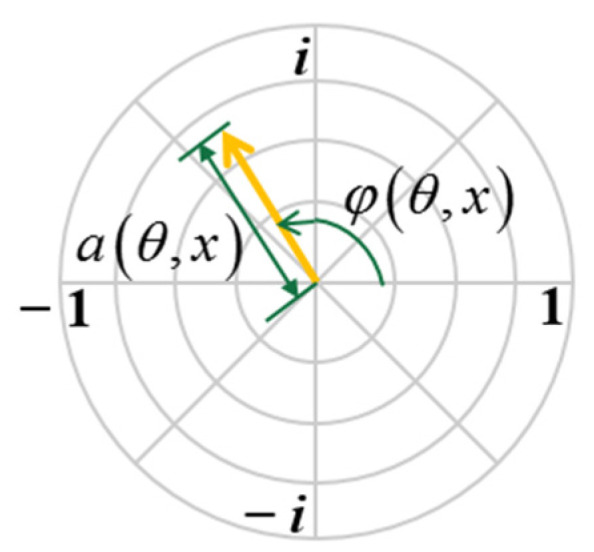
Polarization phasor representation.

The phasor representation of measurements from a ToF camera is proposed in [26] and further developed in [27], which comprehensively analyzes the information of the amplitude and depth. Based on the current studies, we applied the phasor representation in ToF polarization measurements (Figure 4), and expressed as

(1) $$p(\theta ,x)=a\left(\theta ,x\right)e^{i\varphi \left(\theta ,d\left(x\right)\right)}$$

where x represents an exact pixel; θ is the angle between the orientation of linear polarizer and the orientation of the linear polarization analyzer; a is the amplitude at pixel x; φ is the phase difference between the sent and the reflected light; and φ(θ,d(x))=4πfd(x)/c; d(x) is the distance between the target and the sensor at pixel x. The co-linear image is obtained when the orientation of the analyzer is identical to that of the linear polarizer, which are represented as $p(\theta_{\left|\right|},x)$. The cross-linear image $p(\theta_{⊥},x)$ is captured with the orientation of the analyzer orthogonal to that of the linear polarizer.

We assume that the target radiance is unpolarized, the scattering medium is spatially homogeneous, and the single scattering is dominated, as with many earlier works [33,34,35,36,38,39,40,41]. Based on the above assumption, the amplitude is invariant at different θ, and the phase is related to the actual distance and the modulation frequency of the light source, in a clear scene. Therefore, $p(\theta_{\left|\right|},x)=p(\theta_{⊥},x)$, and $p(\theta_{\left|\right|},x)$ coincides with $p(\theta_{⊥},x)$ in the polar coordinate.

**Figure 5:**
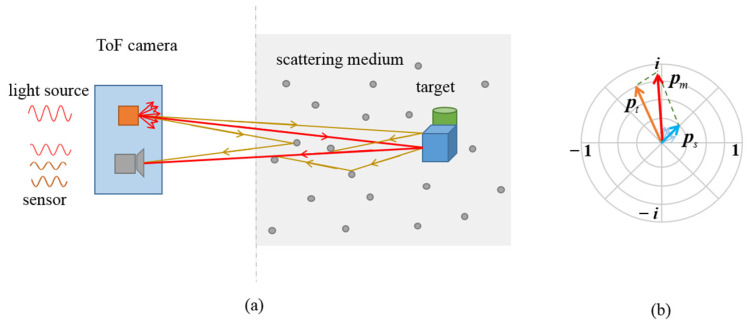
The imaging process and phasor representation of CW-ToF imaging in fog. (**a**) Light path of ToF imaging in the scattering scene. The red lines are the optical paths of directly reflected photons, and the brown lines represent the light paths of scattered photons. (**b**) Phasor representation of ToF measurements in fog. The observed phasor in red can be decomposed into the target phasor in orange and the scattering phasors in blue.

When the scattering medium exists in a scene, the signal received by ToF cameras is not the single-path reflected light from the scene, but the summation of the directly reflected light and multipath scattered light. This phenomenon is contrary to the principle of the ToF measurement, so significant errors are induced. Figure 5 shows the process of ToF imaging in fog, and the corresponding phasor representation. Assuming that the scattering medium is spatially uniform, the observed phasor is the composition of the phasors from the target and scattering components, as shown in Figure 5b, and expressed as

(2) $$\begin{array}{cc} p_{m}\left(\theta ,x\right) & =p_{t}\left(\theta ,x\right)+p_{s}\left(\theta ,x\right)\\ & ={\tilde{a}}_{t}\left(\theta ,x\right)e^{i\varphi \left(\theta ,d\left(x\right)\right)}+\int_{L\left(d\left(x\right)\right)}s\left(\theta \right)e^{i\varphi \left(\theta ,d\left(l\right)\right)}dl \end{array}$$

where ${\tilde{a}}_{t}$ is the attenuated amplitude of the target component, and ${\tilde{a}}_{t}=\mu \cdot a_{t}$; μ is the transmittance of the scattering medium, and $a_{t}$ is the amplitude of the target in clear scenes; s(θ) is the amplitude of the scattering component; and L(d(x)) is the summation of all possible optical paths in front of the target.

### 2.2. Polarization Phasor Imaging Model

According to the theory of optical polarimetric defogging [33], the observed phasors measured at different orientations of the analyzer can be expressed as

(3) $$p_{m}\left(\theta_{\left|\right|},x\right)=p_{t}\left(\theta_{\left|\right|},x\right)+p_{s}\left(\theta_{\left|\right|},x\right)$$

(4) $$p_{m}\left(\theta_{⊥},x\right)=p_{t}\left(\theta_{⊥},x\right)+p_{s}\left(\theta_{⊥},x\right)$$

where $p_{t}\left(\theta_{\left|\right|},x\right)$ and $p_{s}\left(\theta_{\left|\right|},x\right)$ are phasors of the target and scattering components measured at $\theta_{\left|\right|}$; $p_{t}\left(\theta_{⊥},x\right)$ and $p_{s}\left(\theta_{⊥},x\right)$ are phasors of the target and scattering components measured at $\theta_{⊥}$. In addition, the total measurement is the vector summation of data measured at two orthogonal polarization states, which is

(5) $$\begin{array}{c} p_{m}\left(x\right)=p_{m}\left(\theta_{\left|\right|},x\right)+p_{m}\left(\theta_{⊥},x\right)\\ p_{t}\left(x\right)=p_{t}\left(\theta_{\left|\right|},x\right)+p_{t}\left(\theta_{⊥},x\right)\\ p_{s}\left(x\right)=p_{s}\left(\theta_{\left|\right|},x\right)+p_{s}\left(\theta_{⊥},x\right) \end{array}$$

where $p_{t}\left(\theta_{\left|\right|},x\right)=p_{t}\left(\theta_{⊥},x\right)=\frac{1}{2}p_{t}$, since the amplitude of the target component is invariant when the orientation of the analyzer changes, and the phase of the target component is independent on the orientation of the analyzer.

Here, we define the degree of polarization phasor (DOPP) of the observed and scattering phasors as $PP_{m}$ and $PP_{s}$, respectively, and they are represented as

(6) $$\begin{array}{l} PP_{m}=\frac{p_{m}\left(\theta_{\left|\right|},x\right)\;-\;p_{m}\left(\theta_{⊥},x\right)}{p_{m}\left(\theta_{\left|\right|},x\right)\;+\;p_{m}\left(\theta_{⊥},x\right)}\\ PP_{s}=\frac{p_{s}\left(\theta_{\left|\right|},x\right)\;-\;p_{s}\left(\theta_{⊥},x\right)}{p_{s}\left(\theta_{\left|\right|},x\right)\;+\;p_{s}\left(\theta_{⊥},x\right)} \end{array}$$

**Figure 6:**
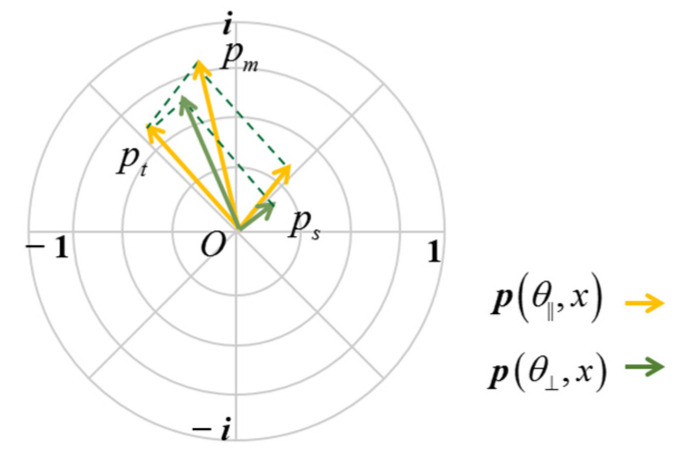
Polarization phasor representation in a scattering scene.

We assume that the proportion of scattering component is higher in the observed measurements measured at $\theta_{\left|\right|}$ than that measured at $\theta_{⊥}$. In this case, $a_{m}\left(\theta_{\left|\right|},x\right)>a_{m}\left(\theta_{⊥},x\right)$, and $a_{s}\left(\theta_{\left|\right|},x\right)>a_{s}\left(\theta_{⊥},x\right)$, as shown in Figure 6. If $\varphi_{s}\left(\theta_{\left|\right|},x\right)=\varphi_{s}\left(\theta_{⊥},x\right)$, $PP_{s}$ is a constant and same as the degree of polarization. Combining the above equations, the scattering phasor can be calculated from

(7) $$p_{s}=\frac{p_{s}\left(\theta_{\left|\right|},x\right)-p_{s}\left(\theta_{⊥},x\right)}{PP_{s}}=\frac{p_{m}\left(\theta_{\left|\right|},x\right)-p_{m}\left(\theta_{⊥},x\right)}{PP_{s}}$$

It can be observed from Equation (7) that $PP_{s}$ is a critical factor to estimate the scattering component. In an outdoor scene, the images captured by a ToF camera usually contain a background, where the measurements can be approximated as the scattering component, since the reflected light is negligible. At this time, $PP_{s}$ can be estimated from the background:

(8) $$PP_{s}=\frac{p_{s}\left(\theta_{\left|\right|},x_{b}\right)-p_{s}\left(\theta_{⊥},x_{b}\right)}{p_{s}\left(\theta_{\left|\right|},x_{b}\right)+p_{s}\left(\theta_{⊥},x_{b}\right)}$$

where $x_{b}$ is the pixel in the background region. After estimating $PP_{s}$, $p_{s}$ can be calculated from Equation (7), and the target component can be obtained from the observed phasor:

(9) $$p_{t}=p_{m}-p_{s}$$

At this time, the depth is recovered from the phase of $p_{t}$ and calculated as

(10) $$d_{recovery}=\frac{c\cdot \arg(p_{t})}{4\pi f},$$

where arg(⋅) operator is used to calculated the angle of the phasor. In the background region, the recovered depth is messy due to the weak reflected light, which is usually suppressed using an image mask [21,22,27]. The image mask can be obtained by image segmentation, and we implemented the maximum between-class variance method [42] on the recovered amplitude image to mask the background of the depth image.

The amplitude recovered from $p_{t}$ is ${\tilde{a}}_{t}$, which contains the attenuation effect of fog. In the traditional optical imaging, the transmittance μ is usually obtained from the scattering component. However, it is not suitable for ToF imaging, since the amplitude of the scattering phasor is affected by the phase. Therefore, image enhancement can be used here to further improve the quality of the amplitude image, such as histogram-based [43,44], Retinex-based [45,46], and grayscale transformation [47,48]. In this work, we used a simple grayscale power transformation from [49] to enhance the amplitude image.

## 3. Experiments and Results

**Figure 7:**
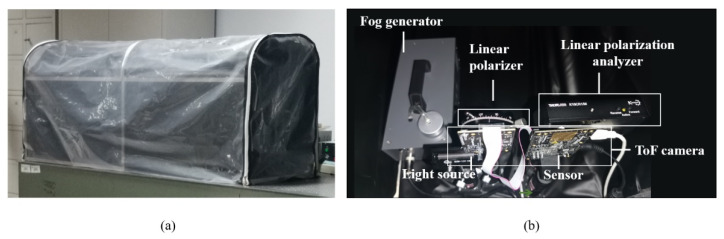
Experimental environment for polarization phasor imaging. (**a**) Fog chamber. Experiments with fog are conducted in this fog chamber. (**b**) Experimental setups.

The effectiveness of the proposed method was evaluated by several experiments with artificial fog. The experiment was implemented in a closed chamber with a light-absorbing cover to avoid additional MPI. The size of the closed chamber is around 120 × 40 × 50 cm^3^, as shown in Figure 7a. Figure 7b shows our experimental setup, which includes a CW-ToF camera manufactured by Texas Instruments (OPT8241-CDK-EVM), a linear polarizer in front of the light source, a linear polarization analyzer in front of the sensor, and a fog generator. The fog was generated from the fog generator by combining water, glycerol and alcohol compounds, and the images were captured after a few minutes, when a stable and uniform scattering environment was formed. The thickness of fog was measured with a laser haze detector (HK-B5S), and described as the number of scattering particles per cubic decimeter. The modulation frequency of the light source was 40 MHz, and the illumination power was 8800 mW, in the parameter settings of the ToF camera. A group of polarization images was captured at two orthogonal orientations of the linear polarization analyzer, with the integration time of 6.2 ms. In addition, the image was averaged from 100 frames for pre-processing to reduce time-dependent noise. The amplitude and depth images captured without fog were considered as the ground truth. The background pixels $x_{b}$ were selected from the area of the light-absorbing cover behind the targets, and the DOPP was estimated by averaging the calculation of the background pixels. The background pixels refer to the distant points and dark points of the scene, whose amplitudes are significantly lower than other pixels, and phases are larger than other pixels.

**Figure 8:**
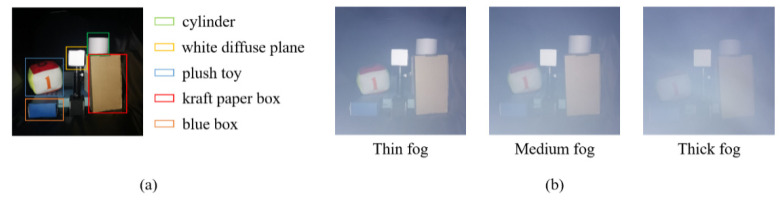
Experimental scene. (**a**) Target objects. (**b**) Scenes under different thicknesses of fog. The numbers of scattering particles per cubic decimeter are 753,640, 935,340, and 1,204,890 from left to right.

**Figure 9:**
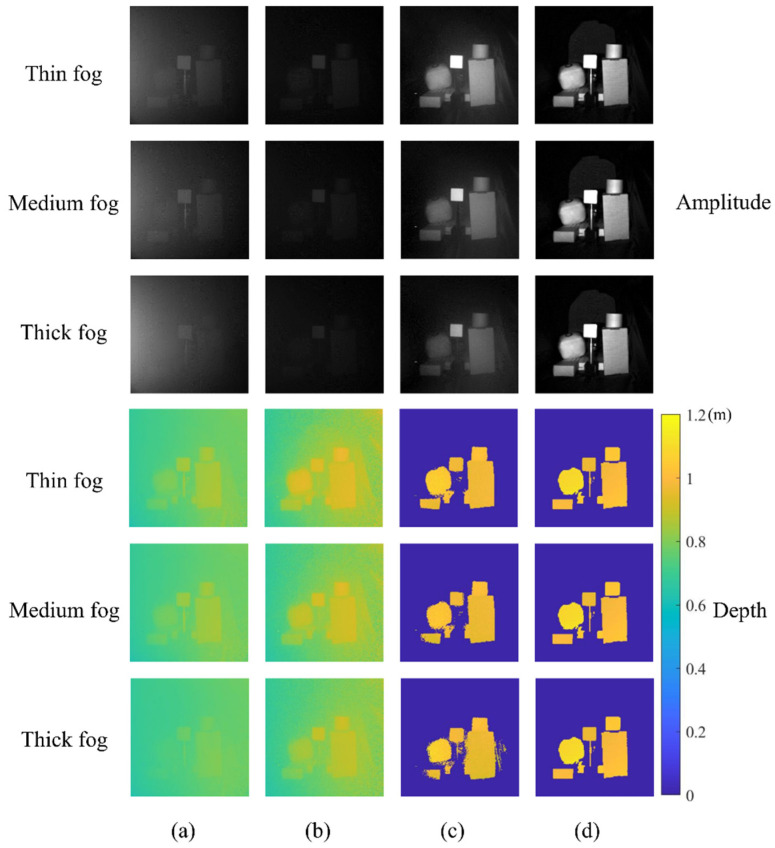
Experimental results in different thicknesses of fog. (**a**) The image captured by an ordinary ToF camera at $\theta_{\left|\right|}$. (**b**) The image captured by an ordinary ToF camera at $\theta_{⊥}$. (**c**) The image recovered by our method. (**d**) The ground truth captured without fog.

The experiment scene is shown in Figure 8a, which contains five targets: a cylinder made of white paper, white diffuse plane, plush toy, kraft paper box, and blue box. The distance between the targets and the sensor was roughly 1 m. We tested our method under different thicknesses of fog (Figure 8b), and the numbers of scattering particles per cubic decimeter are 753,640, 935,340 and 1,204,890, corresponding to thin, medium, and thick fog, respectively. The experiment results are shown in Figure 9, and the quantitative evaluations of the amplitude and depth images are listed in Table 1 and Table 2, respectively.

Ordinary ToF cameras directly capture the amplitude and depth images through the linear polarization analyzer, as shown in Figure 9a,b. The amplitude measured at $\theta_{\left|\right|}$ is brighter than that measured at $\theta_{⊥}$, since the sensor receives more fog components at $\theta_{\left|\right|}$. The depth measured at $\theta_{⊥}$ is closer to the ground truth than that measured at $\theta_{\left|\right|}$ for the same reason. As the density of fog increases, the contrast of the amplitude gradually decreases, and the difference between the measured depth and the ground truth increases. Our method performs well in different thicknesses of fog, as shown in Figure 9c. The contrast of amplitude images is enhanced by our method, and the fog component is effectively suppressed. Meanwhile, the amplitude images are evaluated by the peak signal-to-noise ratio (PSNR) and structural similarity (SSIM) in Table 1. In both indices, our method is superior to the ordinary ToF camera with the linear polarization analyzer.

**Figure 10:**
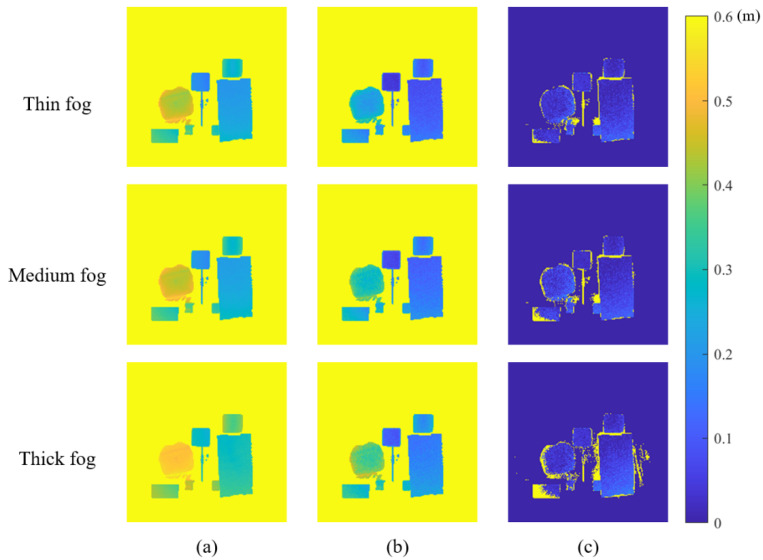
Absolute error of depth under different thicknesses of fog. (**a**) The absolute error of the image captured by an ordinary ToF camera at $\theta_{\left|\right|}$. (**b**) The absolute error of the image captured by an ordinary ToF camera at $\theta_{⊥}$. (**c**) The absolute error of the image recovered by our method.

The depth is greatly distorted in a foggy scene, and it is significantly underestimated using an ordinary ToF camera without other processes, whereas our method restores the depth of different targets in different thicknesses of fog. Figure 10 shows the absolute error of depth under different thickness of fog, and the quantitative results of depth at different target regions are shown in Table 2. In the region of the white diffuse plane and cylinder, the error of depth recovery is the smallest, since the target reflectance and the depolarization effect are the highest. In the edge of the blue box region, some depth information is lost, and the size of depth-loss area increases as the density of the fog increases. This is because the returned light intensity is low, causing the edge to be incorrectly classified as the background during masking background. In addition, flying pixels exist in the recovered depth, especially in thick fog. The reason for this phenomenon is that the background in the recovered amplitude image contains some background information and residual scattering information, and it is easily misclassified when masking background.

## 4. Discussion

### 4.1. The Depolarization Degree of Targets

We assume that the target radiance is unpolarized when ToF polarization imaging in fog. In practice, some target radiance is partial polarization light. In this case, the polarization of the target component should be considered and the scattering phasor is calculated as

(11) $$p_{s}=\frac{\left[p_{m}\left(\theta_{\left|\right|},x\right)-p_{m}\left(\theta_{⊥},x\right)\right]-\left[p_{t}\left(\theta_{\left|\right|},x\right)-p_{t}\left(\theta_{⊥},x\right)\right]}{PP_{s}}$$

Once the scattering phasor is calculated by Equation (7), the estimation error increases. However, the target phasor is unknown and expected to be recovered from the observed phasor, so this is an ill-posed problem. Some studies have been implemented to solve this problem in RGB images [37]. In the future work, we will consider the polarization contribution from the target and improve the estimation accuracy of the scattering phasor.

### 4.2. The Attenuation Factor of the Amplitude

In the computer vision, the transmittance of the scattering medium is usually calculated based on an atmospheric degradation model. However, it is not applicable to directly estimate the transmittance μ from the scattering component for ToF imaging, as the amplitude of the scattering phasor is not a simple superposition of the amplitude in total scattering paths. The transmittance estimation is important to improve the quality of the recovered amplitude image, as well as the image mask. In addition, the transmittance is related to the distance, so calculating the attenuation factor based on the recovered depth information is worth studying in future works.

### 4.3. The Homogeneity of Scattering Media

In this work, the scattering medium is assumed to be spatially homogeneous. In a real foggy environment, atmospheric aerosols are usually non-uniformly distributed in space, which are usually non-uniformly distributed in the direction vertical to the ground and homogeneously distributed in the direction parallel to the ground. The proposed method can estimate the scattering component caused by fog per pixel, so the assumption is not the limitation of our method in most foggy scenes.

## 5. Conclusions

We proposed a polarization phasor imaging method for recovering the depth and amplitude information using the polarization ToF imaging system. We established a polarization imaging model and defined DOPP, which was used to estimate the scattering component for image recovery. The effectiveness of the proposed method was verified by several experiments with different thicknesses of fog. However, this method faced several challenges, such as the low depolarization degree of targets, obtaining the attenuation factor of amplitudes, and non-uniform scattering media. In the future research, the polarization degree of targets and the attenuation factor should be considered to enhance the applicability in practical scenes.

## Figures and Tables

**Table 1:** Quantitative evaluation of the amplitudes using PSNR and SSIM.

Thickness of Fog	Amplitude	PSNR (dB)	SSIM
Thin	$\mathrm{Ordinary}\;\mathrm{ToF}\;\mathrm{measured}\;\mathrm{at}\;\theta_{\left|\right|}$	59.48	0.338
	$\mathrm{Ordinary}\;\mathrm{ToF}\;\mathrm{measured}\;\mathrm{at}\;\theta_{⊥}$	57.92	0.262
	Ours	**63.75**	**0.684**
Medium	$\mathrm{Ordinary}\;\mathrm{ToF}\;\mathrm{measured}\;\mathrm{at}\;\theta_{\left|\right|}$	59.47	0.328
	$\mathrm{Ordinary}\;\mathrm{ToF}\;\mathrm{measured}\;\mathrm{at}\;\theta_{⊥}$	57.87	0.249
	Ours	**61.40**	**0.583**
Thick	$\mathrm{Ordinary}\;\mathrm{ToF}\;\mathrm{measured}\;\mathrm{at}\;\theta_{\left|\right|}$	59.21	0.294
	$\mathrm{Ordinary}\;\mathrm{ToF}\;\mathrm{measured}\;\mathrm{at}\;\theta_{⊥}$	57.84	0.239
	Ours	**60.28**	**0.504**

**Table 2:** Quantitative evaluation of depths using the mean absolute error and mean squared error (MSE). Each cell shows the mean absolute error (m)/MSE.

Thickness of Fog	Depth	Cylinder	White Diffuse Plane	Plush Toy	Kraft Paper Box	Blue Box
Thin	$\mathrm{Ordinary}\;\mathrm{ToF}\;\mathrm{measured}\;\mathrm{at}\;\theta_{\left|\right|}$	0.28/3.03	0.18/1.74	0.43/6.35	0.21/2.80	0.34/3.06
	$\mathrm{Ordinary}\;\mathrm{ToF}\;\mathrm{measured}\;\mathrm{at}\;\theta_{⊥}$	0.11/1.27	0.05/0.45	0.22/3.23	0.09/1.21	0.21/2.30
	Ours	**0.02/0.18**	**0.01/0.14**	**0.03/0.57**	**0.03/0.38**	**0.04/0.49**
Medium	$\mathrm{Ordinary}\;\mathrm{ToF}\;\mathrm{measured}\;\mathrm{at}\;\theta_{\left|\right|}$	0.28/2.52	0.19/1.50	0.44/6.58	0.23/3.75	0.35/5.32
	$\mathrm{Ordinary}\;\mathrm{ToF}\;\mathrm{measured}\;\mathrm{at}\;\theta_{⊥}$	0.14/1.30	0.07/0.55	0.29/4.55	0.12/2.05	0.27/3.29
	Ours	**0.02/0.20**	**0.02/0.17**	**0.04/0.60**	**0.03/0.53**	**0.05/0.66**
Thick	$\mathrm{Ordinary}\;\mathrm{ToF}\;\mathrm{measured}\;\mathrm{at}\;\theta_{\left|\right|}$	0.37/3.29	0.28/2.80	0.52/6.94	0.30/4.84	0.40/5.38
	$\mathrm{Ordinary}\;\mathrm{ToF}\;\mathrm{measured}\;\mathrm{at}\;\theta_{⊥}$	0.19/1.68	0.10/1.04	0.35/4.73	0.16/2.66	0.30/4.07
	Ours	**0.03/0.23**	**0.03/0.28**	**0.06/0.67**	**0.06/0.98**	**0.05/0.70**

## Data Availability

Not applicable.
